# Long-Term Outcomes in Patients with Incident Chronic Obstructive Pulmonary Disease after Acute Kidney Injury: A Competing-Risk Analysis of a Nationwide Cohort

**DOI:** 10.3390/jcm7090237

**Published:** 2018-08-24

**Authors:** Che-Hsiung Wu, Huang-Ming Chang, Cheng-Yi Wang, Likwang Chen, Liang-Wen Chen, Chien-Heng Lai, Shuenn-Wen Kuo, Hao-Chien Wang, Vin-Cent Wu, The National Taiwan University Study Group on Acute Renal Failure

**Affiliations:** 1 Division of Nephrology, Taipei Tzu Chi Hospital, Buddhist Tzu Chi Medical Foundation, New Taipei City 231, Taiwan; tcubear@gmail.com; 2 School of Medicine, Tzu Chi University, Hualien 970, Taiwan; 3 Department of Internal Medicine, National Taiwan University Hospital, Taipei 100, Taiwan; eddie925@ms33.hinet.net (H.-M.C.); haochienwang@gmail.com (H.-C.W.); 4 Department of Internal Medicine, College of Medicine, Fu Jen Catholic University, New Taipei City 242, Taiwan; cywang@mospital.com; 5 Institute of Population Health Sciences, National Health Research Institutes, Zhunan 350, Taiwan; likwang.chen@gmail.com; 6 Department of Surgery, National Taiwan University Hospital, National Taiwan University, Taipei 100, Taiwan; liangwenyumin@gmail.com (L.-W.C.); b8805007@gmail.com (C.-H.L.); shuenn8@gmail.com (S.-W.K.)

**Keywords:** acute kidney injury, chronic obstructive pulmonary disease, congestive heart failure, stroke

## Abstract

Both acute kidney injury (AKI) and chronic obstructive pulmonary disease (COPD) are associated with increased morbidity and mortality. However, the incidence of de novo COPD in patients with AKI, and the impact of concurrent COPD on the outcome during post-AKI care is unclear. Patients who recovered from dialysis-requiring AKI (AKI-D) during index hospitalizations between 1998 and 2010 were identified from nationwide administrative registries. A competing risk analysis was conducted to predict the incidence of adverse cardiovascular events and mortality. Among the 14,871 patients who recovered from temporary dialysis, 1535 (10.7%) were identified as having COPD (COPD group) one year after index discharge and matched with 1473 patients without COPD (non-COPD group) using propensity scores. Patients with acute kidney disease superimposed withs COPD were associated with a higher risk of incident ischemic stroke (subdistribution hazard ratio (sHR), 1.52; 95% confidence interval (95% CI), 1.17 to 1.97; *p* = 0.002) and congestive heart failure (CHF; sHR, 1.61; (95% CI), 1.39 to 1.86; *p* < 0.001). The risks of incident hemorrhagic stroke, myocardial infarction, end-stage renal disease, and mortality were not statistically different between the COPD and non-COPD groups. This observation adds another dimension to accumulating evidence regarding pulmo-renal consequences after AKI.

## 1. Introduction

The incidence of acute kidney injury (AKI) in hospitalized patients is increasing [1] and has been associated with high mortality and morbidity worldwide over the past decade [2]. The incidence of AKI requiring dialysis (AKI-D) is increasing by 10% per year in the United States and is higher than that of end-stage renal disease (ESRD) [3]. Patient survival from an episode of AKI has been improved by advances in critical care medicine and dialysis technology increasing the survival rate of hospitalized patients discharged after temporary dialysis [4]. Previous studies showed that patients with a history of AKI have a higher incidence of coronary events [5], stroke [6], congestive heart failure (CHF) [7], ESRD, and mortality [8] than individuals without AKI. The American Society of Nephrology AKI Advisory Group has highlighted the transition of care as a potential opportunity to reduce the long-term impact of AKI [9]. To improve the situation of dialysis patients, novel renal replacement therapies, such as an implantable artificial renal assist device, are under development. The artificial renal assist device strategy utilizes micromachining techniques to fabricate a biohybrid system able to mimic renal morphology and function [10]. Chronic obstructive pulmonary disease (COPD) is a chronic inflammatory lung disease characterized by airflow limitations. Comorbidities are common in COPD, including cardiovascular [11], cerebrovascular [12], and chronic kidney diseases [13]. These comorbidities are possibly attributed to a chronic inflammatory state in COPD and are increasingly recognized as important determinants of COPD prognosis [13].

Each injured organ can initiate various complex pathways affecting distant organs through hemodynamic, neurohormonal, and cell signaling feedback mechanisms [14]. The kidney plays a key role in fluid, electrolyte, acid-base and clearance homeostasis so that AKI provides a significant impulse for the initiation of organ crosstalk. At the cellular level, the renal tubular epithelium plays a fundamental role in regulating the inflammatory processes [7]. A study has shown that lung inflammation is a consistent finding after ischemic AKI [15]. Nonetheless, there is no study addressing the incidence of COPD in patients with AKI-D. In addition, metabolic and respiratory acidosis is a common and severe complication observed in patients with AKI-D and COPD. When COPD occurs in patients with renal failure, the compensatory role of the kidneys and lungs in acidosis may be less effective, resulting in a more severe acidosis status. The recent Kidney Disease Improving Global Outcomes (KDIGO) guideline introduced a new conceptual model, called acute kidney disease (AKD), to emphasize the need to follow patients who survived AKI episodes. The AKD period, linking AKI to chronic kidney disease (CKD), requires intensive care to manage possible hypertension and cardiovascular disease [16]. However, no study has ascertained the contributing role of COPD in patients with a history of AKI in aggravating subsequent morbidity and mortality. With the increasing recognition that COPD and kidney disease extend beyond the pulmo-renal syndrome, interest in lung–kidney–cardiovascular interactions has increased. Using the Taiwan National Health Insurance research dataset, we designed a nationwide, population-based cohort study to examine the long-term risk of adverse cardiovascular incidents, chronic dialysis events, and mortality in patients with COPD during the AKD period.

## 2. Methods

### 2.1. Data Sources

This population-based cohort study used medical information from Taiwan’s National Health Insurance (NHI) database, a compulsory universal health insurance program that covers outpatient visits, hospital admissions, prescriptions, interventions, disease profiles, and vital status of nearly all 23.7 million Taiwan residents. The NHI database is one of the largest and most comprehensive health-care registries worldwide. Patients were anonymous in our study; thus, informed consent was not required. Additionally, since the identification numbers of all individuals in the database were encrypted to protect their privacy, this study was exempt from a full ethical review by the National Taiwan University Hospital institutional review board (201212021RINC).

### 2.2. Study Group 

The first AKI-D after hospitalization was defined as the index hospitalization for each individual. AKI was defined using the International Classification of Diseases-9 (ICD-9) codes 584.X, 634.3, 635.3, 636.3, 637.3, 638.3, 639.3, 669.3, and 958.5 and procedure codes for acute dialysis. Dialysis certificate data in Taiwan are highly reliable because they are used for insurance payments [17]. We extracted all newly diagnosed AKI patients with de novo AKI-D (identified using the procedure codes) during their index hospitalizations who subsequently recovered from AKI-D (dialysis withdrawal) at least 30 days after discharge between 1 January 1998 and 31 December 2010. Pre-admission comorbidities were identified during at least three outpatient visits or during one inpatient claim within 1 year preceding the index admission. In Taiwan’s National Health Insurance Research Database, ICD-9 codes are used to define diseases. We excluded individuals who had a previous diagnosis of AKI; received a kidney transplant; undergone creation of hemodialysis vascular accesses, peritoneal dialysis catheter implantation, or any form of dialysis preceding the index hospitalization; hospitalizations >180 days with AKI; or those who died during the index hospitalization. Our study enrolled only patients who survived the index hospitalization and had no re-dialysis 30 days after discharge. This identification procedure avoided selective bias [18,19].

**Figure 1:**
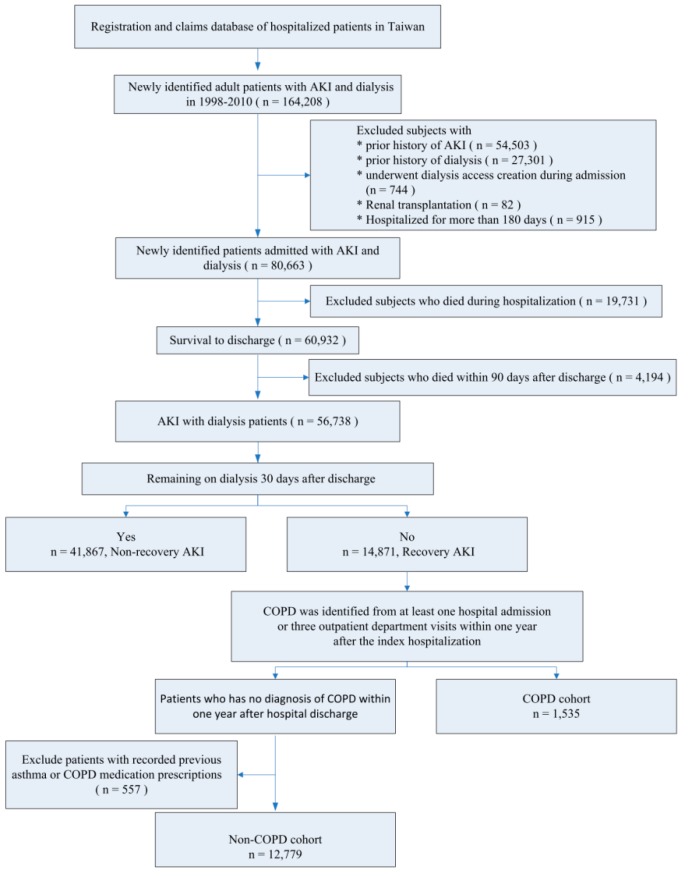
Flow Chart of the enrollee.

The COPD group comprised patients with AKI who recovered from dialysis and incidental COPD within 1 year after the index hospitalization. To ensure accuracy, the diagnosis of COPD was validated based on one inpatient or three outpatient records with ICD-9-CM codes 490–492, 494, and 496 [20,21] and received at least one bronchodilator during the follow-up period [22]. Patients without COPD and no recorded previous asthma or COPD medication prescriptions were included in the control (non-COPD) group (Figure 1).

### 2.3. Baseline Characteristics

The baseline comorbidities were identified from at least three outpatient visits or one inpatient claim within one year preceding the index hospitalization. This identification method has been well validated with good predictive power [18,23,24,25,26]. The Charlson comorbidity index (CCI) was calculated by weighting baseline comorbidities. We collected concomitant medication data associated with the outcomes of interest. According to the Taiwan NHI reimbursement policy, erythropoiesis-stimulating agents may only be prescribed for pre-dialysis chronic kidney disease (CKD) patients with anemia, hematocrits ≤28%, and serum creatinine levels >6 mg/dL (equivalent to an estimated glomerular filtration rate of <15 mL/min/1.73 m^2^, CKD stage 5). We defined this combination of CKD diagnosis codes and pre-dialysis patients using erythropoiesis-stimulating agents as having “advanced CKD” [27]. 

### 2.4. Outcomes 

In order to avoid immortal time bias, the observation period began one year after the index hospitalization discharge and continued until the first documented outcome of interest or the end of the study (31 December 2010), whichever occurred first. The outcomes of interest included all-cause mortality, hospitalization, or death with a principal diagnosis of ischemic stroke (ICD-9-CM code 433.x, 434.x, or 436), hemorrhagic stroke (ICD-9-CM code 431 or 432) [6], CHF (ICD-9-CM code 428.x), major adverse cardiovascular events (MACE), and ESRD [28]. 

Stroke was defined as one of the following conditions [29]: (a) records of emergency room service or hospitalization claims for >1 day or records of emergency room service with ICD-9-CM codes followed by claims for various brain-imaging technologies (computed tomography, magnetic resonance imaging, transcranial or carotid Doppler sonography) or claims for rehabilitation and anti-coagulation prescriptions customarily used for ischemic stroke; or (b) records of three or more consecutive outpatient visits with the above codes and claims for examinations, services, or prescriptions as described in (a) [24]. A reproducibility study found that the ICD-9 stroke codes from the Taiwan National Health Research Institutes at hospital discharge were highly accurate with a substantial kappa test [26]. Furthermore, prior studies have shown that the algorithms using ICD-9 diagnostic codes have a positive predictive value (>95%) for heart failure hospitalizations [26,30].

MACE included nonfatal myocardial infarction (ICD-9-CM code 410.x) [26], coronary artery bypass grafts, and percutaneous transluminal coronary angioplasty [31]. Records of coronary artery bypass grafts and angiography were reliable because they were constructed based on NHI procedure codes that were tied to the audited NHI reimbursement system. In Taiwan, patients who continue dialysis for >90 days receive a catastrophic illness registration card, which ensures the accuracy of our dialysis continuation definition.

### 2.5. Statistical Analysis

Continuous variables were compared using an unpaired t-test and are expressed as a mean ± standard deviation (SD). Categorical variables were compared using the χ^2^ test and expressed as a percentage. 

Given the differences in baseline characteristics and the risk of cardiovascular disease between the incident COPD and non-COPD groups, we matched COPD patients to non-COPD patients using a greedy matching algorithm with a caliper width of 0.2 SDs of the log of the odds of the estimated propensity score with a 1:1 ratio (Supplementary Table S1). Crude hazard ratios (HR) with 95% confidence intervals (CIs) for the outcomes of interest were derived from Cox proportional hazards models. Matched individuals without COPD constituted the reference group. Because of the high mortality rate in patients with COPD after AKI-D, competing risk regression was also performed using the Fine and Gray model considering the subdistribution hazard [32,33]. We used R software version 2.8.1 (Free Software Foundation, Inc., Boston, MA, USA) for the time-varying Cox model and Stata/MP version 14 (Stata Corporation, Lakeway Drive College Station, TX, USA) for the competing risk analysis. Two-sided *p* values <0.05 were considered statistically significant.

## 3. Results

### 3.1. Characteristics of the Study Population

A total of 14,871 individuals after short-term dialysis who survived after hospital discharge were eligible. Among these patients, incident COPD was identified in 1535 (10.7%); 557 with recorded previous asthma or COPD medication prescriptions were excluded. The remaining 12,779 patients were non-COPD controls (Figure 1). The mean age of the COPD group was 73.91 ± 11.25 years, and the proportion of men was 66.94%. After propensity score matching, we identified 1473 patients with COPD and 1473 matched controls with similar baseline characteristics. Detailed demographic information of the individuals with or without COPD before and after propensity score matching is shown in Table 1 and Supplementary Figure S1.

### 3.2. Long-Term Risks of Death, Stroke, and CHF

After a mean follow-up period of 3.32 years, a total of 1050 (71.28%) and 971 (65.92%) patients in the COPD and the non-COPD groups, respectively, died (Table 2). The incidences of all-cause mortality were 226.6 per 1000 person-years in the COPD group and 188.2 per 1000 person-years in the non-COPD group (Table 3). The disparity in all-cause mortality between the two groups was not statistically significant after adjusting the propensity score and renal function status during the AKD period (adjusted HR, 1.04; 95% CI, 0.96–1.14; *p* = 0.331).

**Figure 2:**
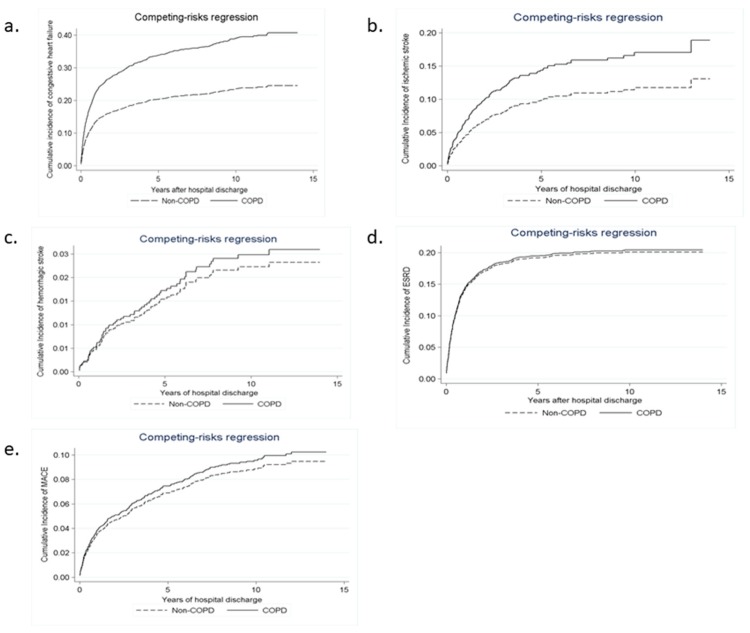
Cumulative probability of (**a**) congestive heart failure (HR 1.61; *p* < 0.001), (**b**) ischemic stroke (HR 1.52; *p* = 0.002), (**c**) hemorrhagic stroke (HR 1.19; *p* = 0.480), (**d**) end- stage renal disease (HR 0.95; *p* = 0.550), and (**e**) major adverse cardiovascular events (HR 1.09; *p* = 0.520) in the matched patients after acute kidney injury with or without chronic obstructive pulmonary disease, using mortality as a competing risk.

In contrast to the non-COPD group, the COPD group had a higher long-term risk of incident CHF (sHR, 1.61; 95% CI, 1.39–1.86; *p* < 0.001; Figure 2a) and ischemic stroke (sHR, 1.52; 95% CI, 1.17–1.97; *p* = 0.002; Figure 2b), but had a similar risk of hemorrhagic stroke (sHR, 1.19; 95% CI, 0.73–1.96; *p* = 0.480; Figure 2c) after considering mortality as a competing risk. 

### 3.3. Long-Term Risks of MACE and ESRD 

A total of 95 (6.45%) patients in the COPD group and 87 (5.91%) in the non-COPD group experienced MACE, but the difference was not statistically significant (Table 2; *p* = 0.592). The COPD group had similar risks of ESRD compared with the matched non-COPD group (*p* = 0.927). After adjusting for in-hospital death as a competing risk, the analysis yielded consistent results (Figure 2d,e). The subdistribution hazard ratio for MACE was 1.09 (95% CI, 0.84–1.40; *p* = 0.520) and 0.95 (95% CI, 0.81–1.12; *p* = 0.550) for ESRD (Table 3).

### 3.4. Subgroup Analysis with Comorbidities

**Figure 3:**
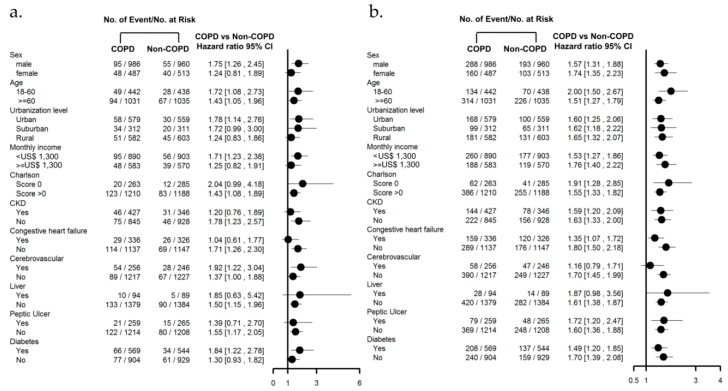
Risk of (**a**) ischemic stroke, and (**b**) congestive heart failure in matched patients after AKI with or without COPD using participant characteristics.

A subgroup analysis of baseline characteristics and comorbidities was performed to investigate whether the COPD group consistently had a higher long-term risk of ischemic stroke and CHF compared to the non-COPD group. Using participant characteristics, we found that COPD was associated with a higher risk of ischemic stroke and CHF in most patients after AKI regardless of any prior history of COPD (Figure 3a,b).

## 4. Discussion

In this large population-based group study, we found that more than one-tenth of patients were likely to have COPD early after temporary dialysis. In patients who recovered from AKI-D, concomitant COPD was associated with a higher risk of incident ischemic stroke and CHF; however, the risks of all-cause mortality, MACE, and ESRD were similar to those who did not have COPD. These results remained constant even after the identification of severe kidney sequelae during the AKD period and when accounting for death as a competing risk.

### 4.1. Risk of COPD after AKI-D

The current study revealed that 10.7% of patients who recovered from temporary dialysis were concomitantly diagnosed with COPD one year after index hospitalization. To our knowledge, this is the first study addressing the incidence of COPD after AKI-D. The age-standardized reported prevalence of COPD was 3.2% in males and 2.0% in females [34]. A population-based study demonstrated that the diagnosis of COPD was recorded in 2.6% of hospitalized patients [35]. The incidence of COPD in patients with AKI-D is much higher than in the general population. Data from animal models support the supposition that cardiogenic pulmonary edema and non-cardiogenic pulmonary edema (from endothelial injury due to inflammation and apoptosis) can occur in AKI [36]. Lung inflammation is a consistent finding after ischemic AKI, especially after prolonged AKI with more than 7 days of dialysis [15]. Accumulated evidence from animal models and patients with AKI suggests that IL-6, IL-8, TNF, and caspase-3-mediated apoptosis are mediators of lung injury after AKI [36,37,38]. These cytokines play an important role in many immunopathological processes of COPD [39,40] and could lead to the development of COPD [40,41].

### 4.2. Stroke Risk in Patients with COPD after AKI-D

Our results further showed that patients with COPD after recovery from AKI-D, compared to those without COPD, have an approximately 52% increased risk of ischemic stroke. According to the results of our specificity analysis (Figure 3a), the incidence is especially high for male patients and for those with diabetes. 

In patients with COPD, systemic inflammation secondary to pulmonary inflammation can elicit unstable atherosclerotic plaques and a pro-thrombotic status, with an eventual ischemic stroke [42,43]. Our findings raise the possibility that in AKI patients, COPD may further trigger a cascade of perturbations that never completely resolve. Some nontraditional risk factors such as endothelial dysfunction, impaired endothelial progenitor cells, oxidative stress, and inflammation during AKI may be involved in the pathogenic mechanisms of lung–kidney–brain interactions [44,45,46]. Acid–base disturbances in patients with COPD after AKI-D may also contribute to the increased risk of ischemic stroke. Acidemia fully protonates free fatty acids forming an oil phase that may fuse with the endothelium and initiate plaque formation [47]. Respiratory acidosis could further serve as a risk factor for thrombus formation [48] and involves the accumulation of serum calcium and phosphate ions which has a major influence on the vascular endothelium [49].

Soluble Klotho could exert multiple actions, including anti-oxidation, anti-senescence, autophagy, anti-apoptosis, and anti-fibrosis [50]. AKI is a state of acute Klotho deficiency. Klotho deficiency exerts multiple negative systemic effects on numerous organs including the cardiovascular system [51]. Importantly, Klotho expression was also reduced in lung alveolar macrophages and peripheral blood mononuclear cells of COPD patients [52]. The enhanced deficiency of Klotho in AKI patients superimposed with COPD will further lead to impaired endothelium-dependent vasodilation and impaired angiogenesis and is related to ischemic stroke [53] and cardiomyopathy [54]. 

### 4.3. CHF Risk in Patients with COPD after AKI-D

Our results also showed that patients with COPD after recovery from AKI-D have an approximately 61% increased risk of CHF compared with those without COPD. AKI itself could cause a number of systemic vascular endothelial alterations that impact cardiovascular health [55]. In type 3 cardiorenal syndrome, AKI can lead to cardiac dysfunction by fluid overload, electrolytes, acid–base shift, and renin-angiotensin-aldosterone system or central nervous system activation [7]. Among COPD patients, the prevalence of ventricular dysfunction was 12–17% [56,57]. Patients with COPD were more likely to develop new-onset heart failure during their hospital stay [58]. In addition, pulmonary hypertension is common in severe COPD and can lead to heart failure. Chronic severe hypoxia, on the other hand, increases plasma norepinephrine and aldosterone, but suppresses renin activity and causes salt and water retention in humans [59]. When COPD occurs in AKI-D patients, the compensatory role of the kidney and lung in acidosis is less effective, resulting in a more severe acidosis state which is known to reduce left ventricle contractility [60]. These are possible mechanisms by which COPD confers additional risks to CHF among AKI survivors.

Human neutrophil gelatinase-associated lipocalin (NGAL), initially identified as a protein isolated from the secondary granules of human neutrophils, is actively secreted by certain cells such as respiratory epithelial cells and renal tubule cells [61]. It was reported to be an important player in vascular remodeling, atherosclerotic plaque stability, and thrombus formation [62]. NGAL from neutrophils may drive COPD epithelial mesenchymal transitions [63] and could reflect the state of systemic inflammation in COPD. In light of this, plasma NGAL which supposedly accumulates in AKI patients with COPD during the AKD period, could serve as a predictor of stroke [64] and heart failure [65].

### 4.4. Risk of MI and ESRD in Patients with COPD after AKI-D

AKI as well as COPD increases the risk of MI [5,66]. However, our study showed that the incidence of MI was not further increased in AKI-D patients with COPD, as compared to those without COPD. This finding implies that shared risk factors of AKI and COPD accounts for much of the elevated risk, and that COPD does not confer a large additional risk. Similarly, previous research suggested that AKI is associated with an increased risk of ESRD [3]. Since AKI already has a strong association with MACE and ESRD, the presence of COPD adds little attributable risk of MACE and ESRD in the nationwide population. 

### 4.5. Care of Patients with COPD after AKI-D

Clinicians should be alert to the presence of COPD among patients with AKI-D. AKI and COPD are now global health problems; however, the AKD period has not been listed as a clinically important consequence in clinical guidelines of lung diseases [67]. Findings of the current study are noteworthy from the perspective of a clinician caring for a patient with COPD after recovery from AKI-D. Attention should be given to the importance of raising awareness about the co-existence of COPD with AKI and cardiovascular risks. A public health initiative is needed to monitor and control subsequent adverse cardiovascular events, including ischemic stroke and CHF, among AKI-D patients with COPD especially after discharge. Optimizing the control of respiratory and uremic conditions, early detection of cardiovascular complications, and decreasing inflammatory status may be the best strategies for improving the quality of health care. Additionally, the pathophysiologic link between kidney and lung disease deserves further investigation, particularly during AKD care.

### 4.6. Limitation and Strength 

A few clinical and research implications emerged from our study. The NHI database has a large national sample size, a long follow-up, and we used a propensity score method to reduce imbalances in key characteristics between COPD and non-COPD groups. The ICD-9-CM codes and procedure codes for AKI and COPD were well validated. Although smaller studies would need to rely on changes in estimated glomerular filtration rates or albuminuria as kidney end points, the availability of incident ESRD, major adverse cardiac events, and mortality as primary outcomes in this study is a notable strength. However, the present study has some limitations that should also be acknowledged. First, like all claims databases, the data describing lifestyle factors such as body mass index and smoking are not available, and residual confounding may be contributing to the association of COPD with outcomes. However, these missing confounding factors were adjusted by obesity and smoking-related disorders such as hyperlipidemia, hypertension, and ischemic heart disease. Second, the NHI research database does not contain information on several potential confounders, including nutritional conditions, proteinuria data, and the adequacy of glycemic, lipid, and blood pressure control. Finally, certain medications used by COPD patients may serve as potential confounders. For example, combined inhalers, containing a long-acting β2-agonist and an anticholinergic, when compared to monotherapy, were associated with an increased risk of heart failure [68].

## 5. Conclusions

In a large cohort study, more than one-tenth of the patients who recovered from AKI-D were diagnosed with COPD at their one-year follow-up. Adverse cardiovascular events including CHF and ischemic stroke are more prevalent in these patients; however, the risk of ESRD, myocardial infarction, or mortality are not different in patients with or without COPD. A public health initiative is needed to monitor and control subsequent adverse cardiovascular events among COPD patients during the AKD period, even those who have recovered from temporary dialysis.

## Figures and Tables

**Table 1:** AKI patients after temporary dialysis with and without COPD, before and after propensity score matching.

	Before Matching			After Matching		
	Non COPD	COPD	*p*-Value	Non COPD	COPD	*p*-Value
	(*n* = 12,779)	(*n* = 1535)		(*n* = 1473)	(*n* = 1473)	
Age (year, SD)	63.63 ± 16.39	74.02 ± 11.31	<0.001	73.95 ± 11.28	73.91 ± 11.25	0.888
Male gender	6715 (52.55%)	1044 (68.01%)	<0.001	960 (65.17%)	986 (66.94%)	0.331
Monthly income, US dollars						
<600	7865 (61.55%)	923 (60.13%)	0.004	903 (61.30%)	890 (60.42%)	0.304
600–1300	4484 (35.09%)	581 (37.85%)		530 (35.98%)	554 (37.61%)	
≥1300	430 (3.36%)	31 (2.02%)		40 (2.72%)	29 (1.97%)	
Hospital location						
Urban	5276 (41.29%)	604 (39.35%)	0.005	559 (37.95%)	579 (39.31%)	0.696
Suburban	2947 (23.06%)	320 (20.85%)		311 (21.11%)	312 (21.18%)	
Rural	4556 (35.65%)	611 (39.80%)		603 (40.94%)	582 (39.51%)	
Baseline comorbidities						
Charlson comorbidity index	2.17 ± 2.03	2.61 ± 2.06	<0.001	2.59 ± 2.09	2.61 ± 2.08	0.736
Myocardial infarction	542 (4.24%)	75 (4.89%)	0.232	68 (4.62%)	75 (5.09%)	0.607
Congestive heart failure	1956 (15.31%)	349 (22.74%)	<0.001	326 (22.13%)	336 (22.81%)	0.691
Peripheral vascular disease	244 (1.91%)	25 (1.63%)	0.487	29 (1.97%)	25 (1.70%)	0.681
Cerebrovascular disease	1382 (10.81%)	276 (17.98%)	<0.001	246 (16.70%)	256 (17.38%)	0.659
Dementia	315 (2.46%)	98 (6.38%)	<0.001	91 (6.18%)	91 (6.18%)	0.999
Reumatologic disease	243 (1.90%)	18 (1.17%)	0.043	16 (1.09%)	18 (1.22%)	0.863
Peptic ulcer disease	1791 (14.02%)	273 (17.79%)	<0.001	265 (17.99%)	259 (17.58%)	0.81
Hemiplegia or paraplegia	117 (0.92%)	26 (1.69%)	0.006	16 (1.09%)	26 (1.77%)	0.161
Diabetes	4956 (38.78%)	582 (37.92%)	0.524	544 (36.93%)	569 (38.63%)	0.362
Moderate or severe liver disease	1036 (8.11%)	98 (6.38%)	0.019	89 (6.04%)	94 (6.38%)	0.76
Chronic kidney disease	4034 (31.57%)	445 (28.99%)	0.041	447 (30.35%)	437 (29.67%)	0.718
Hypertension	6630 (51.88%)	950 (61.89%)	<0.001	889 (60.35%)	911 (61.85%)	0.427
Dyslipidemia	1765 (13.81%)	167 (10.88%)	0.001	185 (12.56%)	164 (11.13%)	0.254
Medication for hypertension before index hospitalization						
Alpha-blocker	1326 (10.38%)	206 (13.42%)	<0.001	201 (13.65%)	201 (13.65%)	0.999
Beta-blocker	4696 (36.75%)	509 (33.16%)	0.006	507 (34.42%)	494 (33.54%)	0.641
CCB	6807 (53.27%)	946 (61.63%)	<0.001	881 (59.81%)	903 (61.30%)	0.429
Diuretic	6657 (52.09%)	926 (60.33%)	<0.001	859 (58.32%)	887 (60.22%)	0.311
ACEI or ARB	5577 (43.64%)	767 (49.97%)	<0.001	719 (48.81%)	744 (50.51%)	0.376
Other medication						
Aspirin	1107 (8.66%)	163 (10.62%)	0.013	171 (11.61%)	156 (10.59%)	0.412
Clopidogrel	640 (5.01%)	107 (6.97%)	0.002	97 (6.59%)	105 (7.13%)	0.61
Ticlopidine	471 (3.69%)	76 (4.95%)	0.017	70 (4.75%)	74 (5.02%)	0.798
Dipyridamole	2851 (22.31%)	357 (23.26%)	0.4	358 (24.30%)	348 (23.63%)	0.698
Nitrate	93 (0.73%)	22 (1.43%)	0.006	19 (1.29%)	19 (1.29%)	0.999
Statin	2009 (15.72%)	191 (12.44%)	0.001	209 (14.19%)	184 (12.49%)	0.193
NSAID	6375 (49.89%)	859 (55.96%)	<0.001	822 (55.80%)	819 (55.60%)	0.941
PPI	1214 (9.50%)	201 (13.09%)	<0.001	165 (11.20%)	191 (12.97%)	0.158
Index hospital comorbidity						
Cardiovascular	1148 (8.98%)	150 (9.77%)	0.301	153 (10.39%)	142 (9.64%)	0.539
Respiratory	2819 (22.06%)	635 (41.37%)	<0.001	602 (40.87%)	582 (39.51%)	0.475
Hepatic	267 (2.09%)	15 (0.98%)	0.002	22 (1.49%)	14 (0.95%)	0.24
Neurologic	250 (1.96%)	37 (2.41%)	0.247	35 (2.38%)	35 (2.38%)	0.999
Hematologic	201 (1.57%)	16 (1.04%)	0.121	17 (1.15%)	15 (1.02%)	0.859
Metabolic	365 (2.86%)	35 (2.28%)	0.219	41 (2.78%)	35 (2.38%)	0.561
ICU admission	8492 (66.45%)	1189 (77.46%)	<0.001	1141 (77.46%)	1130 (76.71%)	0.661
Operation	1314 (10.28%)	151 (9.84%)	0.624	136 (9.23%)	139 (9.44%)	0.899
Renal function status at AKD periods (1 year after index hospitalization)						
CKD	4958 (38.80%)	642 (41.82%)	0.023	532 (36.12%)	622 (42.23%)	0.001
Advanced CKD	2410 (18.86%)	206 (13.42%)	<0.001	199 (13.51%)	201 (13.65%)	0.957

All data were descripted as number (%), except mean age. Abbreviations: ACEI, angiotensin-converting-enzyme inhibitors; AKD, acute kidney disease; ARB, Angiotensin II receptor blockers; CCB, calcium channel blocker; CKD, chronic kidney disease; COPD, chronic obstructive pulmonary disease; GI, gastrointestinal; ICU, intensive care unit; NSAIDs, Non-steroidal anti-inflammatory drugs; PPI, proton-pump inhibitor; SD, standard deviation.

**Table 2:** Long-term outcomes the first year after index hospitalization discharge.

Events	Before Matching			After Matching		
	Non-COPD (*n* = 12,779)	COPD (*n* = 1535)	*p*-Value	Non-COPD (*n* = 1473)	COPD (*n* = 1473)	*p*-Value
All-cause death	6931 (54.24%)	1096 (71.40%)	<0.001	971 (65.92%)	1050 (71.28%)	0.002
Stroke	1044 (8.17%)	172 (11.21%)	<0.001	121 (8.21%)	170 (11.54%)	0.003
Ischemic stroke	774 (6.06%)	144 (9.38%)	<0.001	95 (6.45%)	143 (9.71%)	0.001
Hemorrhagic stroke	327 (2.56%)	36 (2.35%)	0.668	30 (2.04%)	35 (2.38%)	0.616
CHF	2541 (19.88%)	458 (29.84%)	<0.001	296 (20.10%)	448 (30.41%)	<0.001
MACE *	802 (6.28%)	96 (6.25%)	0.999	87 (5.91%)	95 (6.45%)	0.592
ESRD	3362 (26.31%)	311 (20.26%)	<0.001	299 (20.30%)	302 (20.50%)	0.927

CHF, congestive heart failure; COPD, chronic obstructive pulmonary disease; ESRD, end-stage renal disease; MACE, major adverse cardiovascular events; SD, standard deviation; * MACE includes myocardial infarction, coronary artery bypass graft, and percutaneous transluminal coronary angioplasty.

**Table 3:** Incidence and risk of outcomes of interest among patients after temporary dialysis with and without chronic obstructive pulmonary disease.

	COPD			Non-COPD			Crude		Adjusted ^†^		Compete Risk ^††^	
	Event	Person-Year	Incidence Rate (per 1000 Person-Years)	Event	Person-Year	Incidence Rate (per 1000 Person-Years)	HR (95%CI)	*p* Value	HR (95%CI)	*p* Value	sHR (95%CI)	*p* Value
Before Propensity Score Matching												
All-cause death	1096	4848.71	226.0	6931	57,368.8	120.8	1.55 [1.45,1.65]	<0.001	0.96 [0.90,1.04]	0.323	NA	NA
Stroke	172	4368.39	39.4	1044	53,890	19.4	1.70 [1.45,2.00]	<0.001	1.27 [1.06,1.53]	0.009	1.30 [1.07,1.56]	0.007
Ischemic stroke	144	4413.14	32.6	774	54,369.5	14.2	1.88 [1.57,2.24]	<0.001	1.33 [1.09,1.63]	0.006	1.37 [1.11,1.69]	0.004
Hemorrhagic stroke	36	4783.3	7.5	327	56,658.6	5.8	1.18 [0.84,1.67]	0.349	1.08 [0.74,1.57]	0.691	1.05 [0.72,1.54]	0.8
CHF	458	3769.6	121.5	2541	50,533.8	50.3	1.89 [1.71,2.09]	<0.001	1.37 [1.22,1.53]	<0.001	1.39 [1.24,1.56]	<0.001
MACE	127	4595.71	27.6	1127	54,737.5	20.6	1.20 [1.00,1.44]	0.055	0.89 [0.73,1.09]	0.270	0.89 [0.72,1.08]	0.240
ESRD	311	4073.25	76.4	3362	45,620.2	73.7	0.81 [0.72,0.91]	<0.001	0.90 [0.80,1.02]	0.102	0.91 [0.80,1.03]	0.120
After Propensity Score Matching												
All-cause death	1050	4633.13	226.6	971	5159.11	188.2	1.08 [0.99,1.17]	0.104	1.04 [0.96,1.14]	0.331	NA	NA
Stroke	170	4156.28	40.9	121	4788.18	25.3	1.45 [1.14,1.83]	0.002	1.42 [1.12,1.79]	0.004	1.43 [1.13,1.81]	0.003
Ischemic stroke	143	4201.03	34.0	95	4831.49	19.7	1.52 [1.17,1.97]	0.002	1.48 [1.14,1.92]	0.003	1.52 [1.17,1.97]	0.002
Hemorrhagic stroke	35	4567.72	7.7	30	5107.35	5.9	1.26 [0.77,2.05]	0.362	1.26 [0.77,2.05]	0.361	1.19 [0.73,1.96]	0.480
CHF	448	3583.99	125.0	296	4547.46	65.1	1.62 [1.40,1.88]	<0.001	1.59 [1.37,1.84]	<0.001	1.61 [1.39,1.86]	<0.001
MACE *	126	4391.31	28.7	116	4871.23	23.8	1.13 [0.87,1.45]	0.357	1.12 [0.87,1.44]	0.396	1.09 [0.84,1.40]	0.520
ESRD	302	3870.54	78.0	299	4367.31	68.5	0.97 [0.83,1.14]	0.695	0.96 [0.81,1.12]	0.579	0.95 [0.81,1.12]	0.550

CHF, congestive heart failure; CI, confidence interval; ESRD, end stage renal disease; HR, hazard ratio; MACE, major adverse cardiovascular events, NA, not available; sHR, subdistribution hazard ratio; * MACE includes myocardial infarction, coronary artery bypass graft, and percutaneous transluminal coronary angioplasty. ^†^ Adjusted for propensity score and renal function status 1 year after index hospitalization. ^††^ Death was calculated as a competing risk, adjusted for propensity score and renal function status 1 year after index hospitalization.

## Supplementary Materials

The following are available online at http://www.mdpi.com/2077-0383/7/9/237/s1, Figure S1: Standardized difference between each covariate before and after propensity score matching, Table S1: Risk factors predicting COPD in AKI patients after temporary dialysis as components in the propensity score.
