# Supplementary material for: Long-Term Outcomes in Patients with Incident Chronic Obstructive Pulmonary Disease after Acute Kidney Injury: A Competing-Risk Analysis of a Nationwide Cohort
Source: J Clin Med. 2018 Aug 24;7(9):237. doi: 10.3390/jcm7090237 (PMC6162866; doi:10.3390/jcm7090237)
Supplement: Supplementary file 1 [file jcm-07-00237-s001.pdf]

Supplementary file

Table S1. The risk factors predicting COPD in AKI patients after temporary dialysis as components in propensity score.

Items	OR	lower 95% CI	upper 95% CI	*P*
**Age**	1.041	1.036	1.047	<0.001
**Male**	1.865	1.646	2.113	<0.001
Baseline comorbidities				
Charlson Comorbidity Index	0.911	0.879	0.945	<0.001
Congestive heart failure	1.219	1.039	1.431	0.015
Cerebrovascular disease	1.448	1.220	1.718	<0.001
Medication for hypertension				
Beta-Blocker	0.859	0.755	0.978	0.021
Index hospital comorbidity				
Cardiovascular	0.735	0.599	0.902	0.003
Respiratory	1.759	1.537	2.012	<0.001
ICU admission during index hospitalization	1.161	1.000	1.348	0.049

Hosmer-Lemeshow goodness of fit [GOF] test P =0.405, AUC= 0.811
Abbreviations: AKI, acute kidney injury; COPD, chronic obstructive pulmonary disease; ICU, intensive care unit.

Figure S1. Standardized difference between each covariate before and after propensity score matching

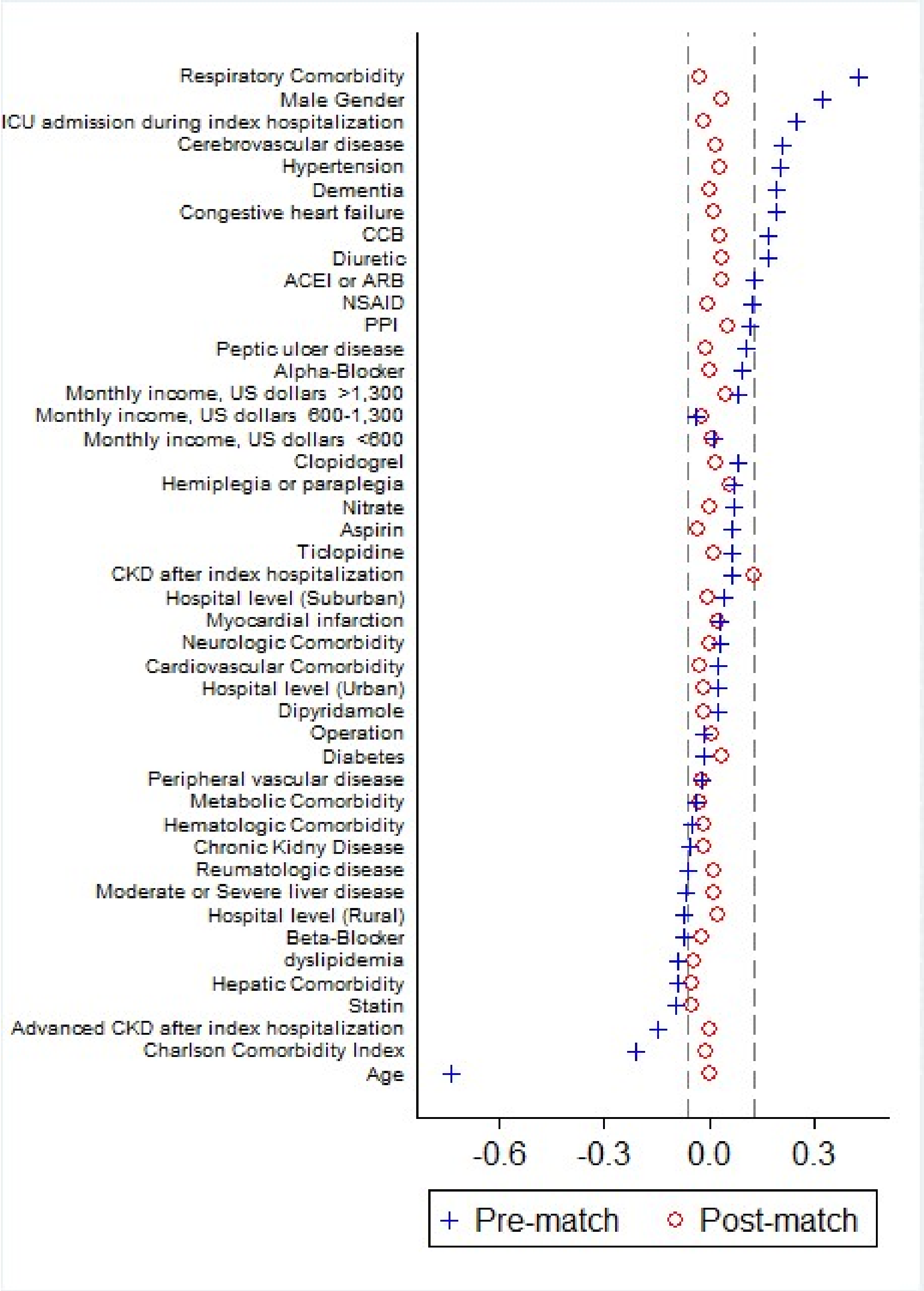

Abbreviations: ACEI, angiotensin-converting-enzyme inhibitors; ARB, Angiotensin II receptor blockers; CCB, calcium channel blocker; COPD, chronic obstructive pulmonary disease; GI, gastrointestinal; ICU, intensive care unit; NSAIDs, Non-steroidal anti-inflammatory drugs; PPI, proton-pump inhibitor; SD, standard deviation
